# CD47 (Cluster of differentiation 47): an anti-phagocytic receptor with a multitude of signaling functions

**DOI:** 10.1080/19768354.2020.1818618

**Published:** 2020-09-15

**Authors:** Pascal Leclair, Chinten James Lim

**Affiliations:** Department of Pediatrics, University of British Columbia, and, Michael Cuccione Childhood Cancer Research Program, B.C. Children’s Hospital Research Institute, Vancouver, Canada

**Keywords:** CD47, phagocytosis, cell death, antibodies, peptides

## Abstract

CD47 is a tumor-associated antigen best known for its ability to bind counter-receptors on the surface of professional phagocytes as an immune-evasion strategy. ⁣Recently, CD47 has been shown to play a role as a signaling receptor, involving a number of cell physiological processes.⁣⁣ This review provides a comprehensive survey of the signaling pathways triggered by CD47 ligand-mediated cell death in tumor cells. Such an understanding should lead to improvement of CD47-targeted anti-tumor therapeutics able to both neutralize the anti-phagocytic role and trigger autonomous tumor cell death.

## CD47 is a ubiquitous cell surface antigen

CD47 was first discovered in 1988 as an unnamed cell-surface glycoprotein that was absent on red blood cells isolated from Rh_null_ syndrome patients, upon investigation with the novel antibody, BRIC 125 (Avent et al. 1988). The next year, another novel antibody, B6H12 – whose antigen was determined to be a heavily glycosylated, cell-surface protein of approximately 50 kDa – was found to functionally and physically associate with β3-integrins; thus, the authors named this protein ‘integrin-associated protein’ (IAP) (Brown et al. 1990). At about the same time, the OA3 antigen – as detected using a third antibody, OVTL3 – was found to be highly expressed on the surface of ovarian carcinomas (Campbell et al. 1992). Cloning of these newly discovered molecules revealed that all three proteins were identical: a protein with an extracellular region containing a human IgV-like domain, five membrane-spanning domains, and a short, alternatively-spliced, cytoplasmic tail (Rebres et al. 2001). In addition, the extracellular domain of CD47 is highly glycosylated and has two disulphide bonds, which ensures proper folding of the extracellular domain to enable ligand binding and transduction of intracellular signaling (Lindberg et al. 1993; Mawby et al. 1994; Reinhold et al. 1995).

**Figure 1.**
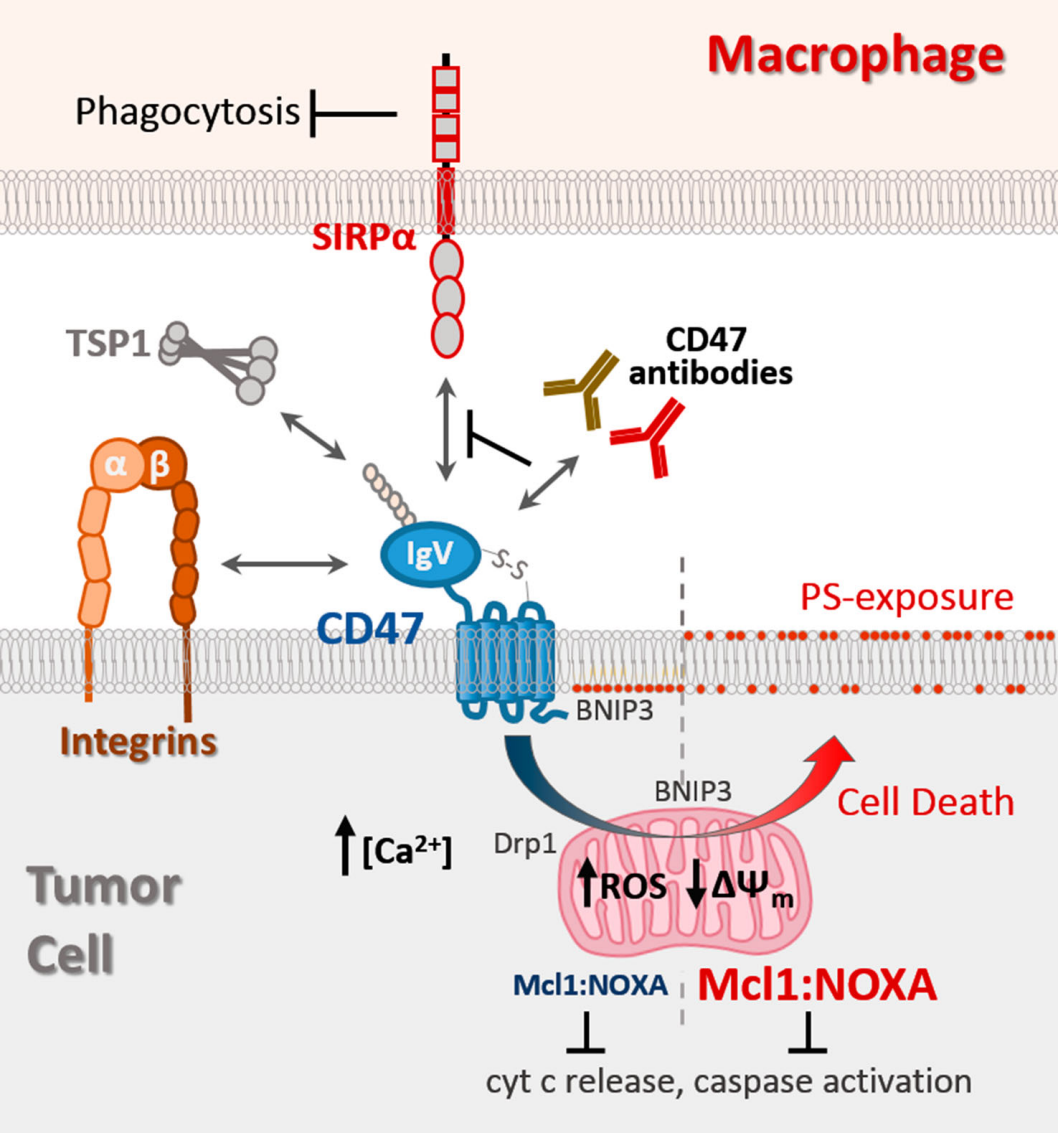
CD47 ligands and transduction of cell death signaling. The globular IgV-like domain of CD47 mediates interactions with soluble matrix protein thrombospondin-1 (TSP1), trans-interactions with SIRPα expressed on macrophages, and cis-interactions with integrins. Antibodies with therapeutic potential can be categorized into ones able to neutralize CD47-SIRPα interaction to relieve the block in phagocytosis (green), and ones able to also induce tumor cell death in a manner not requiring effector cells (red). CD47-ligation induced cell death is accompanied by increases in intracellular Ca^2+^ and reactive oxygen species (ROS), a decrease in mitochondrial membrane potential (ΔΨm), and surface exposure of phosphatidyl serine (PS). CD47-ligation promotes translocation of pro-apoptotic BNIP3 and Drp1 to the mitochondria, as well as inducing concurrent increases in Mcl1 and NOXA protein levels in a manner preventing cytochrome c release and caspase activation.

This review will highlight the known ligands that mediate CD47 receptor signaling, with particular focus on mechanisms that couple CD47-ligation to autonomous cell death (Figure 1).

## CD47 ligands

Thrombospondin (TSP), a component of the extracellular matrix (ECM) with a role in regulating adhesion/motility, angiogenesis during wound healing, and inflammation, was the first protein to be confirmed as a natural ligand for CD47 (Gao et al. 1996; Adams and Lawler 2011). Specifically, a VVM motif at TSP’s C-terminus was found to mediate this interaction, from which the often-used 4N1K, 7N3, and more recently, PKHB1, peptides were subsequently derived and used as proxies for TSP activity (Gao and Frazier 1994; Martinez-Torres et al. 2015). However, it must be noted that these peptides have been conclusively shown to exert a number of biological phenomena independent of their ligand interaction with CD47 (Tulasne et al. 2001; Barazi et al. 2002; Karki and Nichols 2014; Leclair and Lim 2014; Leclair et al. 2020). Therefore, the usage of 4N1K-like peptides in experiments to study CD47 signaling should be carefully considered and critically evaluated. Due to the biologically active, albeit non-specific nature of these peptides, we highly recommend to use adequate controls such as CD47-deficient cells before drawing conclusions regarding CD47-receptor mediated functions.

Arguably, CD47’s claim to fame was the discovery that its interaction with Signal Regulatory Protein α (SIRPα), a counter-receptor that is prominently expressed on macrophages and other professional phagocytic cells, induces ‘don’t eat me’ signaling that protects CD47-expressing cells from phagocytosis (Seiffert et al. 1999; Blazar et al. 2001; Khandelwal et al. 2007; Chao et al. 2019). For example, decreased expression of CD47 on the surface of aged cells facilitates their phagocytic removal, whereas CD47 over-expression on cancer cells constitute an immune-evasion mechanism that confers a pro-survival advantage (Chao et al. 2019). In addition to SIRPα, SIRPγ is an isoform that binds CD47 with a lower affinity, whereas SIRPβ does not bind CD47 to act in anti-phagocytic signaling (Brooke et al. 2004; Chao et al. 2019).

## Cell death induced by CD47-ligation

In 1999, two groups independently discovered an unexpected but important role for CD47, that ligation of CD47 led to rapid induction of cell death in a cell autonomous manner (Mateo et al. 1999; Pettersen et al. 1999). Mateo *et al.* noticed that patient-derived chronic lymphocytic leukemia (CLL) cells showed decreased viability when attached to immobilized B6H12 antibody (Mateo et al. 1999). On the other hand, Pettersen *et al.* reported that CD47 functions as a comitogenic signal in T-cell activation, noticing that ligation of the receptor by anti-CD47 antibody clones 1F7 or Ad22, but not clones B6H12 or 2D3, resulted in morphological changes reminiscent of apoptosis (Pettersen et al. 1999). Upon further investigation, it was determined that ligation of CD47 for as little as 30 min resulted in cytoplasmic signs of apoptosis such as cell shrinkage, exposure of phosphatidylserine (PS) on the outer plasma membrane, loss of mitochondrial membrane potential (ΔΨ_m_), increased reactive oxygen species (ROS), a drop in ATP levels, and the eventual permeabilization of the plasma membrane (Mateo et al. 1999; Pettersen et al. 1999; Mateo et al. 2002; Lamy et al. 2003; Manna and Frazier 2003; Roue et al. 2003; Bras et al. 2007). Following these initial reports, CD47 ligation-mediated cell death was confirmed in tumor cells of lymphoid (Mateo et al. 2002; Leclair et al. 2018), myeloid (Mateo et al. 2002), granulocytes (Mateo et al. 2002), dendritic cells (Johansson et al. 2004), ovaries (Mateo et al. 2002), and breasts (Manna and Frazier 2004). Importantly, CD47 ligation-mediated cell death has been shown to be specific for tumor cells, leaving normal, untransformed cells completely unaffected (Mateo et al. 2002; Martinez-Torres et al. 2015; Uscanga-Palomeque et al. 2019); it should be noted, however, that normal T-cells were shown to be sensitive to CD47-directed treatments when first pre-activated with anti-CD3 antibodies (Manna and Frazier 2003)(further discussed below).

Consistent with the rapid induction of cell death mentioned above, CD47 ligation-mediated cell death was shown to be induced in a manner that did not require transcription or translation (Bras et al. 2007). Furthermore, although this phenomenon was independent of TCR signaling, CD47 ligation-mediated cell death was significantly increased by co-ligation with an anti-CD3 antibody, suggesting a role for T-cell activation in this process (Pettersen et al. 1999; Mateo et al. 2002). As already mentioned, CD47 acts as a ‘don’t eat me’ signal to macrophages and other phagocytes; in contrast, calreticulin, an ER-resident chaperone protein, translocates to the cell surface during ER stress and apoptosis where it acts as the main ‘eat me’ signal for phagocytes, thereby rendering the cell immunogenic (Chao et al. 2019). As such, stimulation of phagocytosis involves the delicate balance between CD47 down-regulation and calreticulin exposure on the cell surface. Using the TSP-derived peptide, PKHB1, Martinez-Torres et al. have shown that PKHB1-mediated cell death was potentially immunogenic by inducing cell surface presentation of damage-associated molecular pattern (DAMP) molecules including calreticulin (Martinez-Torres et al. 2015; Martinez-Torres et al. 2019; Uscanga-Palomeque et al. 2019). However, it remains unclear if PKHB1 stimulation of immunogenic cell death is mediated via ligation of CD47, since both PKHB1 and 4N1K can effectively induce cell death in CD47-deficient leukemic cells (Leclair et al. 2020).

Characterization of the CD47 domains revealed that the transmembrane and extracellular domains of CD47 are required for transduction of cell death-inducing signals. However, the C-terminal cytoplasmic tail seems irrelevant since expression of all four isoforms comparably induced cell death, as did replacement of CD47’s cytoplasmic tail with that of CD7 (Mateo et al. 2002; Leclair et al. 2018). Crosslinking of antibodies was proposed to be required to mediate cell death effects, although this is not the case for antibody clone CC2C6, since it can be used in the soluble form in the absence of secondary antibodies (Mateo et al. 1999; Kikuchi et al. 2004; Leclair et al. 2018). CD47-mediated cell death is likely to be epitope-dependent since antibodies whose epitopes overlap – such as B6H12, BRIC-125, Ad22 and CC2C6 – have cell death-inducing capabilities (though the former two require immobilization), whereas clone 2D3 – which binds CD47 at a distant site from the above-mentioned antibodies – is often used as a control since it does not induce cell death (Pettersen et al. 1999; Manna and Frazier 2003). Surprisingly, co-incubation with 2D3 was found to significantly inhibit CD47-mediated cell death induced by 1F7 and Ad22, which suggests a requirement for a flexible extracellular domain (Pettersen et al. 1999). Importantly, clone CC2C6, but not B6H12, can only bind CD47 containing an N-terminal pyroglutamate, a post-translational modification that is catalyzed by the enzyme glutaminyl cyclase (Hatherley et al. 2008; Logtenberg et al. 2019; Wu et al. 2019). In addition, binding of SIRPα to CD47 also requires the pyroglutamate epitope. It is not yet known if all CD47-antibodies capable of inducing cell death share a common requirement for post-translationally modified CD47 with the N-terminal pyroglutamate.

## Role of T-cell activation in CD47-mediated cell death

It was shown that anti-CD47 antibodies inhibited integrin-mediated phagocytosis, adhesion to several integrin ligands, and fibronectin-mediated Ca^2+^ increases (Gresham et al. 1989; Brown et al. 1990; Schwartz et al. 1993). Despite these indications of CD47 connection with integrins, studies with erythrocytes, which highly express CD47 but no integrins, strongly suggested that CD47 can exert function in a manner independent of integrins. Furthermore, ligation by immobilized B6H12 generated a respiratory burst akin to that resulting from phagocyte activation (Zhou and Brown 1993). As such, a hypothesis was soon proposed that CD47 may act as a comitogenic signal for T-cell activation. Indeed, anti-CD47 antibodies stimulated T-cell activation – as reported by an increase in proliferation, IL-2 secretion, CD25 expression, activation of p56^lck^, and phosphorylation of TCR ζ and ZAP70 – when co-immobilized with an anti-CD3 antibody or co-incubated with PMA (Reinhold et al. 1997; Ticchioni et al. 1997; Waclavicek et al. 1997). Cross-linking was shown to be vital in these studies because antibodies in solution had no effect on T-cell activation.

During T-cell activation, negative regulators limit this process by inducing cell death via a mechanism known as activation-induced cell death (AICD), which is normally mediated by Fas protein. However, Pettersen et al*.* found that this phenomenon could also be mediated by CD47 since incubation of T-cells with their novel anti-CD47 antibody, Ad22, resulted in significant cell death in a manner independent of Fas (Pettersen et al. 1999). Interestingly, a CD47-deficient Jurkat cell line exhibit reduced sensitivity to Fas-mediated apoptosis that was restored upon CD47-reexpression, indicating CD47 enhances Fas-mediated cell death (Manna et al. 2005). Consistent with a role for CD47 in T-cell activation, co-stimulation with an anti-CD3 antibody not only significantly increased CD47-mediated cell death, but also seemed to be required for an efficient response (Pettersen et al. 1999; Manna and Frazier 2003; Lamy et al. 2007). However, this point is contentious as others have found that CD3 ligation conferred a protective effect against CD47-mediated cell death in normal T-cells (Mateo et al. 2002). Finally, in support of a requirement for lymphocyte activation, T-cell-activated B-cells were shown to be significantly more sensitive to CD47-mediated cell death than their non-activated counterpart (Mateo et al. 2002).

## CD47-ligation promotes ‘Type III’ programed cell death

Apoptosis is tightly regulated by the Bcl-2 family of pro-apoptotic and pro-survival proteins, the latter binding to the former to inhibit their activity. As such, inhibition or down-regulation of pro-survival proteins such as Bcl-2 and Mcl-1 results in induction of apoptosis, whereby oligomers of the mitochondrial transmembrane proteins Bax and Bak form channels through which cytochrome *c*, AIF, endo G, and other resident mitochondrial proteins are released into the cytosol. Importantly, cytochrome *c* forms the apoptosome complex with Apaf-1, dATP, and caspase-9, which activates a number of downstream caspases – proteases that play an essential role in apoptosis by cleaving a host of cellular proteins; while AIF and endo G migrate to the nucleus where they induce DNA fragmentation (Galluzzi et al. 2012).

Thus far, investigations into the role of Bcl-2 family proteins in CD47-mediated cell death have been inconclusive. On one hand, it was shown that CD47-ligation had no effect on Bcl-2 expression (Manna and Frazier 2003; Manna and Frazier 2004; Saumet et al. 2005) and Bcl-2 overexpression did not change the sensitivity of Jurkat cells to CD47 ligation (Bras et al. 2007). However, another group found that Bcl-2 over-expression decreased CD47-mediated cell death in the same cell line (Lamy et al. 2003). Similarly, over-expression of other members of the Bcl-2 family, such as Mcl-1, Bax, Bak, Bim, and Bcl-x_L_, had little effect on CD47-mediated cell death (Bras et al. 2007). Nonetheless, we found that ligation of CD47 by antibody clone CC2C6 upregulated the levels of both Mcl-1 and NOXA, thereby protecting mitochondria from permeabilization (Leclair et al. 2018). Consistent with this, overexpression of NOXA significantly increased CD47-mediated cell death, leading us to propose that the maintenance of a high Mcl-1/NOXA ratio during CD47-mediated cell death prevents the onset of apoptosis.

Although many of the phenotypes described in the introduction are consistent with those induced by apoptosis, an important feature of CD47-mediated cell death is that it occurs in a caspase-independent manner. Indeed, research has shown that it proceeds unaffected by the presence of caspase inhibitors, nor does it induce PARP cleavage, a downstream substrate of one of the so-called executioner caspases, caspase 3 (Mateo et al. 1999; Pettersen et al. 1999; Manna and Frazier 2003; Roue et al. 2003; Manna and Frazier 2004; Bras et al. 2007; Leclair et al. 2018). Consistent with this, CD47-mediated cell death was shown to occur in the absence of classical apoptosis phenotypes resulting from caspase activation, such as nuclear fragmentation or cytochrome *c* release from mitochondria (Mateo et al. 1999; Lamy et al. 2003; Manna and Frazier 2003; Manna and Frazier 2004). Given the lack of caspase activation in CD47-mediated cell death, Bras *et al.* investigated the possibility that other proteases were activated in their stead. As such, the authors found that out of a number of different protease inhibitors tested, only a chymotrypsin-like serine protease inhibitor was effective at preventing cell death induced by immobilized B6H12 (Bras et al. 2007). They also placed the action of the protease upstream of mitochondrial changes, since the former inhibited the latter.

Since CD47-mediated cell death was clearly not mediated via classical apoptosis, several investigators have attempted to better assess this phenomenon. As such, apoptosis-inducing factor (AIF) and Endo G, like cytochrome *c*, were shown to be retained in mitochondria upon ligation of CD47; in addition, although it was determined that lysosomes were permeabilized during CD47-mediated cell death, this was attributed to secondary events since treatment with bafilomycin A, an agent that blocks lysosome function, did not prevent CD47-mediated cell death (Bras et al. 2007). In contrast, some studies provided evidence for the role of autophagy in CD47-mediated cell death (Lamy et al. 2003; Kalas et al. 2013). Consistent with this idea, Merle-Beral *et al.* found that the only inhibitor from a large pool of pharmacological inhibitors to efficiently prevent CD47-mediated cell death was 3-methyladenine (3-MA), an inhibitor of an early stage of autophagy by blockade of PI3K (Merle-Beral et al. 2009). However, ultrastructural studies by Bras *et al.* confirmed that CD47-ligation with immobilized B6H12 induced dilation of the Golgi apparatus, redistribution of the ER around the nucleus, and slight mitochondrial swelling – results consistent with other reports – but without presenting characteristics associated with autophagy (Bras et al. 2007).

The Nomenclature Committee on Cell Death (NCCD) has defined and characterized the various modes of cell death (Kroemer et al. 2005; Galluzzi et al. 2009; Galluzzi et al. 2012). Following the initial elucidation of the main morphological (absence of DNA fragmentation, mitochondria involvement without permeabilization) and biochemical (PS exposure, ROS production, collapse of ΔΨ_m_, caspase-independence) phenotypes, CD47-mediated cell death was proposed to be a novel type of cell death referred to as ‘type III’ programed cell death (PCD) or ‘necrosis-like’ PCD, with mitochondrial disruption as a key event in the regulation of the process (Bras et al. 2007). However, although the NCCD has since expanded and clarified the various modes of cell death and introduced several caspase-independent mechanisms (Galluzzi et al. 2012), classifying CD47-mediated cell death nevertheless remains a difficult task: the lack of AIF release and lysosome involvement (Bras et al. 2007) rules out parthanatos and entosis, respectively; the absence of inhibition by necrostatin-1 (Merle-Beral et al. 2009) rules out necroptosis; autophagic cell death and netosis – two modes of cell death dependent on autophagy – remain tentative since autophagy inhibitors blocked CD47-mediated cell death in one study (Merle-Beral et al. 2009) but were ineffective in another (Martinez-Torres et al. 2019). The reported cell cycle arrest conferred by CD47 ligation (Park et al. 2014) may indicate that cell death proceeds via mitotic catastrophe, although p53-negative cell lines (such as Jurkat cells) are sensitive to CD47-mediated cell death. Finally, mitochondrial transition permeability (MTP)-driven necrosis remains an interesting prospect since it is specifically characterized by abrupt changes in mitochondrial ROS generation, excess Ca^2+^, and loss of ΔΨ_m_, and has been shown to be regulated by Drp1; however, it remains unclear whether CD47-ligation induced cell death is mediated by cyclophilin D, a key regulator in this type of cell death (Galluzzi et al. 2018). As such, the precise mode of cell death elicited by CD47 ligation will need to remain a mystery until more evidence can be brought forward.

## Signaling proteins involved in CD47-mediated cell death

Mitochondrial dynamics is controlled by several proteins which mediate fission and fusion of the organelle. Specifically, Dynamin-related protein 1 (Drp1) is a cytosolic GTPase that relocalizes to mitochondria in a process dependent on chymotrypsin-like serine proteases, and binds to the mitochondrial membrane receptor, hFis1 (Pagliuso et al. 2018). There, it oligomerizes to form a spiral which contracts upon GTP hydrolysis, causing constriction, and then fission, of the mitochondrion. Bras *et al.* sought to determine if Drp1 and mitochondria might be involved in CD47-mediated cell death. Indeed, high Drp1 protein and mRNA levels were correlated to susceptibility of CLL patient B-cells to TSP and immobilized B6H12 (Bras et al. 2007). Gain and loss of Drp1 expression studies implicated Drp1 in the collapse of mitochondrial membrane potential and cell death following anti-CD47 antibody treatment. These activities are seemingly independent of Drp1’s GTPase activity, but required hFis1 expression. Indeed, CD47 ligation resulted in Drp1 translocation to the mitochondria (Bras et al. 2007). As such, the authors proposed that CD47 ligation promotes activation of chymotrypsin-like serine proteases and large-scale Drp1 translocation to mitochondria to bind hFis1, leading to the impairment of the electron transport chain, which in turn results in ROS production, the collapse of ΔΨ_m_, ATP loss, changes in mitochondrial structure, and consequently, cell death. It should be noted that another study found that cells cotreated with antimycin A, an inhibitor of the respiratory chain, completely blocked CD47-mediated PS exposure without affecting the decrease in mitochondrial membrane potential, indicating that these processes may be uncoupled (Mateo et al. 2002).

A number of cell lines have been shown to attach and spread to CD47 antibody-coated dishes, suggesting CD47 couples to cytoskeletal reorganization. This provoked investigation on the role of actin in CD47-mediated cell death. Cytochalasin D, an inhibitor of actin polymerization, was shown to inhibit PS exposure, ΔΨ_m_ decreases, and ROS production following CD47-ligation with antibodies (Pettersen et al. 1999; Mateo et al. 2002; Roue et al. 2003). Bras et al*.* showed that treatment with DyIP, an inhibitor of dynamin proteins/cytoskeleton associations, increased mitochondrial Drp1 and CD47-mediated cell death, suggesting cytoskeletal disassembly was required for translocation of Drp1 to mitochondria (Bras et al. 2007). Furthermore, CD47 ligation did not result in cell death of mononuclear cells from patients with Wiskott-Aldrich syndrome, implicating a requirement for Cdc42 – an upstream GTPase for Arp2/3 activation – in CD47-mediated cell death (Mateo et al. 2002). As such, actin rearrangements seem to be a major crossroads for CD47-mediated cell death. Specifically, actin disassembly triggered by CD47-ligation may release Drp1 to relocate to mitochondria and initiate cell death signaling.

Using a yeast two-hybrid system, Lamy *et al.* determined that the pro-apoptosis protein, 19KDa Interacting Protein-3 (BNIP3), interacted with CD47’s C-terminal transmembrane domain (Lamy et al. 2003). The transmembrane domain of BNIP3 was required for binding to CD47, although other regions of BNIP3 may strengthen the association. Like Drp1, BNIP3 translocates from the cytosol to mitochondria upon ligation of CD47 with antibodies, while knockdown of BNIP3 efficiently prevents CD47-mediated cell death (Lamy et al. 2003; Kalas et al. 2011). In addition, CD47-mediated cell death was correlated to BNIP3 expression in both mouse and human cells, and CD3 ligation increased expression of both BNIP3 mRNA and protein, in line with findings that T-cell activation increases sensitivity to CD47-ligation (Lamy et al. 2007; Kalas et al. 2011). Furthermore, the CD47-BNIP3 association may act to shield against BNIP3 degradation by proteasomes, leading to its accumulation in the cytosol (Lamy et al. 2007). Interestingly, cells overexpressing Bcl-2 exhibit decreased CD47-mediated cell death, suggesting a mechanism whereby BNIP3-Bcl-2 interaction at the mitochondrial membrane may sequester BNIP3 from association with CD47 at the plasma membrane, effectively decoupling CD47-mediated cell death (Lamy et al. 2003).

## Miscellaneous phenotypes that accompany CD47-mediated cell death

Early on, a number of integrin-related, CD47-dependent processes were found to be mediated by activation of trimeric G*_i_*-proteins, such as adhesion, mobility, cell spreading, and chemotaxis (Brown and Frazier 2001; Soto-Pantoja et al. 2015). Consistent with this, pre-incubation of Jurkat and primary T-cells with pertussis toxin (PTX), an inhibitor of G*_i_*-family G-proteins, significantly decreased the cell surface exposure of PS and reversed the decrease in ΔΨ_m_ observed upon CD47-ligation (Manna and Frazier 2003; Manna and Frazier 2004). CD47 also impacted cAMP-PKA signaling: CD47-ligation mediated decreases in cAMP levels could be blocked by PTX and restored by pre-incubation with cAMP agonists, such as forskolin or IBMX (Manna and Frazier 2003; Manna and Frazier 2004). PKA is a major downstream kinase activated by cAMP, and it was observed that inhibition of PKA reversed CD47-mediated cell death inhibition induced by cAMP-elevating agonists (Manna and Frazier 2003). These indicate that cAMP-dependent inhibition of CD47 signaling proceeds via an increase in PKA activity, and that activation of G*_i_* is an early event in CD47-mediated cell death signaling.

Consistent with previous reports linking CD47 ligation and Ca^2+^ fluxes (Schwartz et al. 1993; Tsao and Mousa 1995), treatment of cells with the PKHB1 peptide induced increases in intracellular calcium (Martinez-Torres et al. 2015). Indeed, calcium overload was proposed to mediate the effects of PKHB1 since BAPTA-AM – a cytoplasmic Ca^2+^ chelator – and an inhibitor of the mitochondrial Ca^2+^ uniporter, both abrogated the observed mitochondrial dysfunctions and cell death following PKHB1 treatment. However, these effects may not be CD47-mediated, as PKHB1 is known to induce activity in a CD47-independent manner (Leclair et al. 2020). Using the CC2C6 antibody to induce CD47-dependent cell death, we observed significant increases in cytoplasmic Ca^2+^, however CC2C6-induced cell death was not attenuated upon chelation of calcium (Leclair et al. 2018). Thus, the role of Ca^2+^ flux in modulating CD47-dependent cell death signaling remains unclear.

Epidermal growth factor (EGF) receptor expression is correlated with tumor aggression and a poor prognostic outcome in breast cancers. EGF has also been shown to have protective effects against Fas-, TNFα-, TNFβ-, and TRAIL-induced apoptosis, via upregulation of Akt and ERK activity in a variety of cancer cell lines (Manna and Frazier 2004). Similarly, EGF treatment was shown to effectively protect breast cancer cell lines from 4N1K-mediated cell death, which could be restored by co-incubation of EGF receptor inhibitors or anti-EGFR antibodies (Manna and Frazier 2004). In addition, the PI3 K inhibitor wortmannin reversed the protective effect of EGF against 4N1K-mediated cell death, whereas an ERK inhibitor had no effect, suggesting that Akt downregulation may be involved in CD47-mediated cell death. It was posited that CD47-mediated cell death may involve suppressing Akt or PKA phosphorylation of BAD – a BH3-only pro-survival protein – via activation of trimeric G-protein. Finally, a SIRPα-Fc fusion antibody has been shown to inhibit Akt phosphorylation – as well as a downstream substrate, mTOR – upon treatment of non-small cell lung cancer cells *in vitro* and in a xenograft model, which was proposed to trigger the onset of autophagy (Zhang et al. 2017).

The HIF-1α pathway may also be involved in CD47-mediated cell death. Microarray analysis revealed that CD47-ligation led to upregulation of HIF-1α and HIF-regulated genes, including BNIP3 and RTP801, and treatment with HIF-1α siRNA or with an inhibitor completely prevented CD47-mediated cell death in the lymphocytic cell line, MOLT-4 (Sagawa et al. 2011). Interestingly, hypoxia – acting via HIF-1 – directly upregulated CD47 expression in breast cancer cells, while a survey of TCGA database of breast cancers found a positive correlation between CD47 and HIF target gene expression (Zhang et al. 2015). It would be of interest to study if CD47 ligation-induced cell death may be enhanced for tumors in the hypoxic microenvironment.

Interestingly, Park et al*.* found that CD47-ligation induced cell cycle arrest rather than cell death in Epstein–Barr virus-transformed B-cells. Here, CD47-ligation-induced ROS generation disrupted the expression of various cyclins and CDKs via the phosphorylation of p38 MAPK/JNK, ERK and PI3 K/mTOR pathways. In addition, expression was observed for TAp73, a transcription factor involved in apoptosis and cell cycle arrest, and for CHOP and GRP78, proteins involved in ER stress (Park et al. 2014).

## CD47-mediated cell death in *in vivo* models and therapeutics

The discovery that CD47 expression is often increased on the surface of cancer cells, and CD47’s role as a ‘don’t eat me’ signal for phagocytes, have led to significantly heightened interests in the development of neutralizing antibody-based therapeutics targeting the CD47-SIRPα interaction. However, CD47-targeted therapeutics seeking to both neutralize the anti-phagocytic signal and to engage CD47-ligation mediated cell death, such as that mediated by CC2C6 (Leclair et al. 2018), have received lesser attention.

A significant hurdle with any antibody-based therapeutics targeting CD47 is blood hemagglutination, a consequence of high CD47 expression on red blood cells acting as the antibody ‘sink’ (Liu et al. 2015; Petrova et al. 2017). Several strategies have emerged to mitigate this issue: 1) the generation of single-chain antibody fragments which do not contain an Fc fragment (Kikuchi et al. 2004; Uno et al. 2007); 2) the generation of antibodies consisting of a low-affinity CD47-SIRPα moiety fused to a CD20 epitope (Piccione et al. 2016); and 3) repeated low-dose treatment of a SIRPα/IgG fusion protein (Petrova et al. 2017). For example, Kikuchi *et al.* generated a recombinant single-chain, human anti-CD47 antibody (scFv), MABL scFv-15 – which joins the variable heavy-chain with the variable light-chain domains of the antibody using a 15-residue linker – attempting to manipulate the properties of the antibody (Kikuchi et al. 2004; Uno et al. 2007). Indeed, MABL scFv-15 was shown to efficiently induce cell death in the absence of hemagglutination. In addition, this recombinant antibody proved to have significant anti-tumor effects in a human myeloma xenograft model, indicating that it shows promise as a therapeutic. However, the scFv antibody fragment was shown to have a much shorter half-life than the complete antibody, and therefore required much higher concentrations for effective treatment. Attempts at generating a more stable and effective antibody resulted in the generation of MABL scFv-5 (5-amino acid linker), MABL sc(Fv)2 (covalently linked MABL-scFv-15) and finally, S-S diabody (a disulfide linked version of their antibody)(Kikuchi et al. 2005; Sagawa et al. 2011). It should be noted that the latter of these antibodies was reported to induce cytochrome *c* release from mitochondria and chromatin condensation, which were suggested to be phenotypes unique to S-S diabody-induced, CD47-dependent cell death in B-CLL cells.

For their part, Zhang *et al.* generated a recombinant protein consisting of the first extracellular domain of SIRPα linked to an IgG1 Fc fragment, named SIRPαD1-Fc. Given the presence of an Fc fragment, this antibody induced considerable cytotoxicity via antibody-dependent cell-mediated cytotoxicity (ADCC) in the presence of macrophages, while enabling phagocytosis mediated by CD47-blockade (Zhang et al. 2017; Zhang et al. 2018). However, in contrast to CD47-mediated cell death, this antibody induced cell death involving activation of PARP and caspases, suggesting induction of classical apoptosis unlike those mediated by other CD47-specific antibodies, which were caspase-independent. Finally, the authors found that combination treatment of SIRPαD1-Fc and an autophagy inhibitor significantly increased these effects and led to a decrease in tumor weight compared to single treatments in NSCLC and glioblastoma xenograft models.

Of interest, Peluso et al. recently reported the generation of a human, anti-CD47 antibody, SRF231, which could induce both phagocytosis and cell death *in vitro*, in a number of xenograft models, and in tumor-bearing mice, leading to reduced tumor burden (Peluso et al. 2020). However, both the phagocytosis and cell death induced by the antibody was determined to be Fc receptor-dependent (CD32a), an indication that co-ligation of macrophage receptors by this antibody was required. Importantly, SRF231 bound red blood cells without inducing hemagglutination, thus represent a promising therapeutic antibody targeting CD47.

## Closing remarks

The potential for targeting CD47 as an anti-tumor therapy has reached a new level. Although a number of clinical trials taking advantage of CD47 as a ‘don’t eat me’ signal have been undertaken over the years – several of which are currently underway – those exploring CD47 as an inducer of cell death are only beginning to emerge. A distinct boon to the latter focus is the dual activity provided by certain antibodies (e.g. clone CC2C6) able to induce autonomous cell death sans effector cells, while enabling phagocytosis by blocking the ‘don’t eat me’ signals. However, a significant limit to the therapeutic use of CD47 antibodies – whether with single or dual activity – is the hemagglutinatinon effects resulting from erythrocytes acting as the antigen ‘sink’. Strategies to mitigate the hemaglutination effects that retain the ability to bind the desired pro-tumorigenic epitopes of CD47 remain a significant focus for research on any CD47 antibody slated for further development as a therapeutic.

Despite the fact that many aspects of CD47-mediated cell death are now known, still lacking is a precise pathway for its mechanism of action, the elucidation of which would greatly aid in determining the best drugs to use in combination with CD47 therapies. Nonetheless, one aspect that seems clear is that CD47-mediated cell death induces ROS accumulation, a decrease in ΔΨ_m_, and PS exposure at the cell surface; how these events are connected to CD47 at the cell surface is still unknown, though it seems likely that they are mediated by the translocation of Drp1 and/or BNIP3 to the mitochondria, an event which itself seems to be regulated by actin dynamics. Clarity and confidence in our current knowledge are hampered by the fact that for years, research investigating CD47-mediated cell death was led by use of modified thrombospondin-derived peptides – such as 4N1K and PKHB1 – which we now know exert a number of activities that are CD47-independent. Therefore, much of what we presumably know should be systematically re-investigated and confirmed using CD47-specific agents, such as antibodies validated against CD47-deficient cell lines and animal models expressing human CD47.
